# Supplementary material for: The Effect of Chair-Based Exercise on Physical Function in Older Adults: A Systematic Review and Meta-Analysis
Source: Int J Environ Res Public Health. 2021 Feb 16;18(4):1902. doi: 10.3390/ijerph18041902 (PMC7920319; doi:10.3390/ijerph18041902)

International Journal of
Environmental Research and Public Health

MDPI

Systematic Review

The effect of chair-based exercise on physical function in older adults: a systematic review and meta-analysis

Supplementary Table S1: Medline Search Strategy

#1 (seat* ADJ7 exercis*)
#2 (seat* ADJ7 physio*)
#3 (seat* ADJ7 "physical therap*")
#4 (seat* ADJ7 therap*)
#5 (seat* ADJ7 activit*)
#6 (seat* ADJ7 "tai chi")
#7 (seat* ADJ7 taichi)
#8 (seat* ADJ7 yoga)
#9 (seat* ADJ7 "strength training")
#10 (seat* ADJ7 "resistance training")
#11 (seat* ADJ7 "muscle strengthening")
#12 (seat* ADJ7 train*)
#13 (seat* ADJ7 program*)
#14 (chair* ADJ7 exercis*)
#15 (chair* ADJ7 physio*)
#16 (chair* ADJ7 "physical therap*")
#17 (chair* ADJ7 therap*)
#18 (chair* ADJ7 activit*)
#19 (chair* ADJ7 "tai chi")
#20 (chair* ADJ7 taichi)
#21 (chair* ADJ7 yoga)
#22 (chair* ADJ7 "strength training")
#23 (chair* ADJ7 "resistance training")
#24 (chair* ADJ7 "muscle strengthening")
#25 (chair* ADJ7 train*)
#26 (chair* ADJ7 program*)
#27 ("chair based*" ADJ7 exercis*)
#28 ("chair based*" ADJ7 physio*)
#29 ("chair based*" ADJ7 "physical therap*")
#30 ("chair based*" ADJ7 therap*)
#31 ("chair based*" ADJ7 activit*)
#32 ("chair based*" ADJ7 "tai chi")
#33 ("chair based*" ADJ7 taichi)
#34 ("chair based*" ADJ7 yoga)
#35 ("chair based*" ADJ7 "strength training")
#36 ("chair based*" ADJ7 "resistance training")
#37 ("chair based*" ADJ7 "muscle strengthening")
#38 ("chair based*" ADJ7 train*)
#39 ("chair based*" ADJ7 program*)
#40 ((sit OR sitting OR sat) ADJ7 exercis*)
#40 ((sit OR sitting OR sat) ADJ7 exercis*)
#41 ((sit OR sitting OR sat) ADJ7 physio*)
#42 ((sit OR sitting OR sat) ADJ7 "physical therap*")
#43 ((sit OR sitting OR sat) ADJ7 therap*)

#44 ((sit OR sitting OR sat) ADJ7 activit*)
#45 ((sit OR sitting OR sat) ADJ7 "tai chi")
#46 ((sit OR sitting OR sat) ADJ7 taichi)
#47 ((sit OR sitting OR sat) ADJ7 yoga)
#48 ((sit OR sitting OR sat) ADJ7 "strength training")
#49 ((sit OR sitting OR sat) ADJ7 "resistance training")
#50 ((sit OR sitting OR sat) ADJ7 "muscle strengthening")
#51 ((sit OR sitting OR sat) ADJ7 train*)
#52 ((sit OR sitting OR sat) ADJ7 program*)
#53 (("non weight bearing" OR "non weightbearing" OR "nonweight bearing") ADJ7 exercis*)
#54 (("non weight bearing" OR "non weightbearing" OR "nonweight bearing") ADJ7 physio*)
#55 (("non weight bearing" OR "non weightbearing" OR "nonweight bearing") ADJ7 "physical therap*")
#56 (("non weight bearing" OR "non weightbearing" OR "nonweight bearing") ADJ7 therap*)
#57 (("non weight bearing" OR "non weightbearing" OR "nonweight bearing") ADJ7 activit*)
#58 (("non weight bearing" OR "non weightbearing" OR "nonweight bearing") ADJ7 "tai chi")
#59 (("non weight bearing" OR "non weightbearing" OR "nonweight bearing") ADJ7 taichi)
#60 (("non weight bearing" OR "non weightbearing" OR "nonweight bearing") ADJ7 yoga)
#61 (("non weight bearing" OR "non weightbearing" OR "nonweight bearing") ADJ7 "strength training")
#62 (("non weight bearing" OR "non weightbearing" OR "nonweight bearing") ADJ7 "resistance training")
#63 (("non weight bearing" OR "non weightbearing" OR "nonweight bearing") ADJ7 "muscle strengthening")
#64 (("non weight bearing" OR "non weightbearing" OR "nonweight bearing") ADJ7 train*)
#65 (("non weight bearing" OR "non weightbearing" OR "nonweight bearing") ADJ7 program*)
#66 ("low impact" ADJ7 exercis*)
#67 ("low impact" ADJ7 physio*)
#68 ("low impact" ADJ7 "physical therap*")
#69 ("low impact" ADJ7 therap*)
#70 ("low impact" ADJ7 activit*)
#71 ("low impact" ADJ7 "tai chi")
#72 ("low impact" ADJ7 taichi)
#73 ("low impact" ADJ7 yoga)
#74 ("low impact" ADJ7 "strength training")
#75 ("low impact" ADJ7 "resistance training")
#76 ("low impact" ADJ7 "muscle strengthening")
#77 ("low impact" ADJ7 train*)
#78 ("low impact" ADJ7 program*)
#79 OR(1-78)
#80 "random* control* trial*"
#81 "random* control*"
#82 "random* trial*"
#83 "random* control* stud*"
#84 "random* stud*"
#85 rct
#86 "control* trial*"
#87 "control* stud*"
#88 OR (80-87)
#89 #79 AND #88

Supplementary Table S2: Risk of Bias of Included Studies

Study	Randomization process	Deviations from intended interventions	Missing outcome data	Measurement of the outcome	Selection of the reported result	Overall
Baum (2003)	?	+	+	+	+	+
Daniel (2012)	+	+	+	+	+	+
Dean (2017)	+	+	+	+	+	–
Furtado (2016)	–	+	+	+	+	!
Furtado (2020)	?	+	+	+	+	+
Ikai (2017)	+	+	+	+	+	–
Kertapati (2018)	–	+	+	+	+	+
Kim (2015)	+	+	+	+	+	+
Kujasski (2018)	+	+	+	+	+	+
Latham (2003)	+	+	+	+	+	+
Lee (2015)	+	+	+	+	+	+
McMurdo (1993)	+	+	+	+	+	+
McMurdo (2014)	+	+	+	+	+	!
Netz (2007)	?	+	+	+	+	–
Nicholson (1997)	–	+	+	+	+	+
Niemela (2011)	+	+	+	+	+	+
Park (2014)	+	+	+	+	+	+
Park (2016 & 2017a)	+	+	+	+	+	+
Park (2017b)	+	+	+	+	+	+
Park (2019)	+	+	+	+	+	+
Rieping (2019)	–	+	+	+	+	+
Venturelli (2010)	+	+	+	+	+	+
Vogler (2009)	+	+	+	+	+	+
Vogler (2012)	+	+	+	+	+	–
Yao (2019)	–	+	+	+	+	–

+ Low risk
? Some conce
– High risk

Forest Plots for Meta-Analyses

Figure S1: Berg Balance Scale

Study or Subgroup	Intervention Mean	Intervention SD	Intervention Total	Control Mean	Control SD	Control Total	Weight	Mean Difference IV, Random, 95% CI
Baum 2003	0.6	16.9148	11	5.9	15.3	9	1.4%	-5.30 [-19.44, 8.84]
Kim 2015	27	1.1	15	26.8	2	15	60.9%	0.20 [-0.96, 1.36]
Latham 2003	43	22.3165	122	45	21.167	110	8.0%	-2.00 [-7.60, 3.60]
Niemela 2011	51.5	3	26	49.4	6.1	25	27.0%	2.10 [-0.55, 4.75]
Park 2017b	36.23	12.94	13	28.54	13.25	13	2.7%	7.69 [-2.38, 17.76]
**Total (95% CI)**			**187**			**172**	**100.0%**	**0.66 [-1.01, 2.33]**

Heterogeneity: Tau² = 0.84; Chi² = 5.02, df = 4 (P = 0.29); I² = 20%
Test for overall effect: Z = 0.78 (P = 0.44)

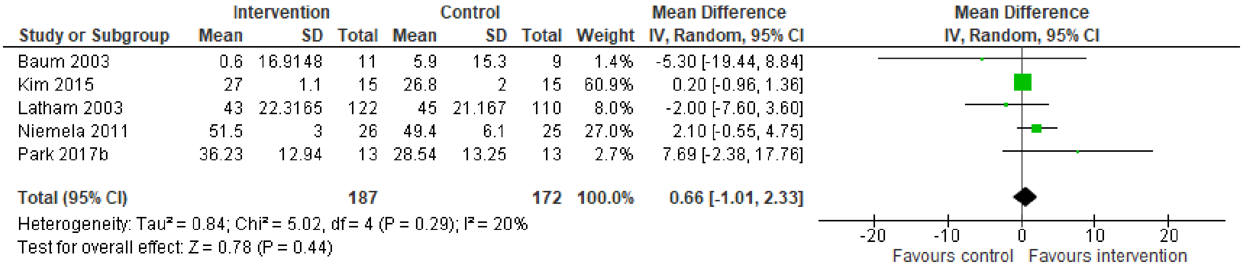

Figure S2: Handgrip Strength

Study or Subgroup	Intervention Mean	Intervention SD	Intervention Total	Control Mean	Control SD	Control Total	Weight	Mean Difference IV, Fixed, 95% CI
Ikai 2017	26.8	9.7	28	25.2	12.5	28	5.2%	1.60 [-4.26, 7.46]
McMurdo 1993	2.2	4.2	15	1	3.6	26	27.6%	1.20 [-1.34, 3.74]
Nicholson 1997	1.15	4.4721	20	-0.49	3.5216	10	20.6%	1.64 [-1.29, 4.57]
Niemela 2011	25	7.6	26	23.7	4.5	25	15.3%	1.30 [-2.11, 4.71]
Park 2017b	21.45	8.65	13	27.62	12.05	13	2.7%	-6.17 [-14.23, 1.89]
Park 2019	22.5433	8.5648	21	21.84	8.64	10	4.2%	0.70 [-5.78, 7.19]
Yao 2019	20.75	3.72	16	15.47	3.94	15	24.3%	5.28 [2.58, 7.98]
**Total (95% CI)**			**139**			**127**	**100.0%**	**2.10 [0.76, 3.43]**

Heterogeneity: Chi² = 10.36, df = 6 (P = 0.11); I² = 42%
Test for overall effect: Z = 3.08 (P = 0.002)

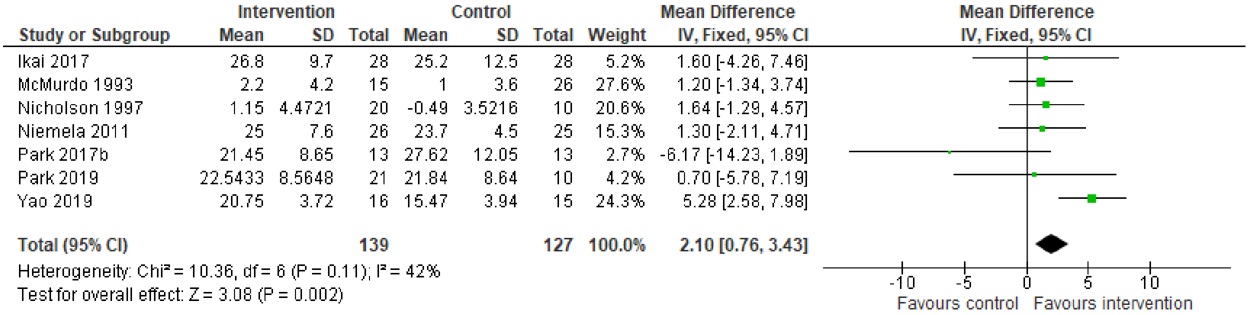

Figure S3: Timed up and Go Test

Study or Subgroup	Intervention Mean	Intervention SD	Intervention Total	Control Mean	Control SD	Control Total	Weight	Mean Difference IV, Random, 95% CI
Baum 2003	18	59.6992	11	-5.5	54	9	0.2%	23.50 [-26.39, 73.39]
Daniel 2012	7.3	1	8	6.4	2	7	35.6%	0.90 [-0.74, 2.54]
Furtado 2016	18.9	12.17	20	14.02	5.75	15	9.2%	4.88 [-1.20, 10.96]
Latham 2003	18	5.3407	112	16	10.5835	110	30.1%	2.00 [-0.21, 4.21]
Netz 2007	18.1	8.8	14	14.7	7.3	10	8.4%	3.40 [-3.06, 9.86]
Park 2019	25.1605	11.0317	21	30.63	17.63	10	2.8%	-5.47 [-17.37, 6.43]
Rieping 2019	12.3738	5.5924	34	16.72	7.87	13	13.7%	-4.35 [-9.02, 0.33]
**Total (95% CI)**			**220**			**174**	**100.0%**	**0.95 [-1.12, 3.01]**

Heterogeneity: Tau² = 2.42; Chi² = 9.83, df = 6 (P = 0.13); I² = 39%
Test for overall effect: Z = 0.90 (P = 0.37)

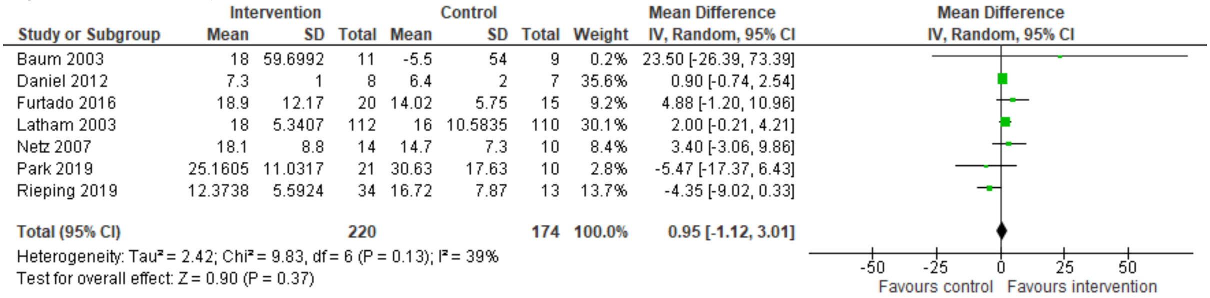

Figure S4: Gait speed (m/s)

Study or Subgroup	Intervention Mean	Intervention SD	Intervention Total	Control Mean	Control SD	Control Total	Weight	Mean Difference IV, Random, 95% CI
Dean 2007	1.07	0.38	5	0.57	0.38	4	9.3%	0.50 [0.00, 1.00]
Kim 2015	0.68	0.1	15	0.92	0.1	15	26.3%	-0.24 [-0.31, -0.17]
Latham 2003	1.5375	0.6008	112	1.425	0.9261	110	20.6%	0.11 [-0.09, 0.32]
Niemela 2011	1.4	0.3	26	1.5	0.4	25	21.1%	-0.10 [-0.29, 0.09]
Park 2016 and 2017a	1.28	0.52	63	1.28	0.55	49	20.8%	0.00 [-0.20, 0.20]
Park 2017b	2.91	1.63	13	3.5	1.78	13	1.9%	-0.59 [-1.90, 0.72]
**Total (95% CI)**			**234**			**216**	**100.0%**	**-0.03 [-0.21, 0.16]**

Heterogeneity: Tau² = 0.03; Chi² = 21.44, df = 5 (P = 0.0007); I² = 77%
Test for overall effect: Z = 0.27 (P = 0.78)

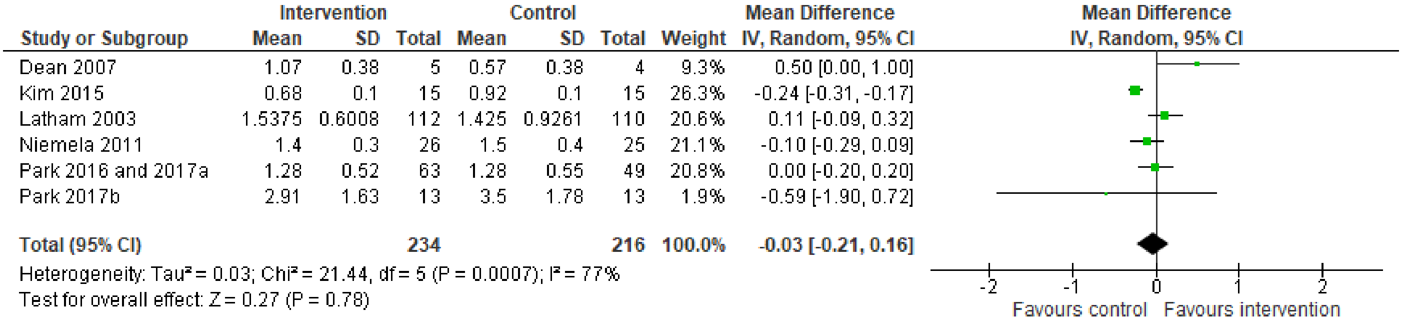

Figure S5: 30 second arm curl test

	Experimental			Control				Mean Difference
**Study or Subgroup**	**Mean**	**SD**	**Total**	**Mean**	**SD**	**Total**	**Weight**	**IV, Fixed, 95% CI**
Daniel 2012	18.1	3.6	8	19.8	3.9	7	15.1%	-1.70 [-5.52, 2.12]
Furtado 2016	10.95	3.33	20	8.06	3.78	15	37.9%	2.89 [0.48, 5.30]
Rieping 2019	11.76	3.67	34	7.54	3.26	13	47.1%	4.22 [2.06, 6.38]
**Total (95% CI)**			**62**			**35**	**100.0%**	**2.82 [1.34, 4.31]**

Heterogeneity: Chi² = 7.00, df = 2 (P = 0.03); I² = 71%
Test for overall effect: Z = 3.74 (P = 0.0002)

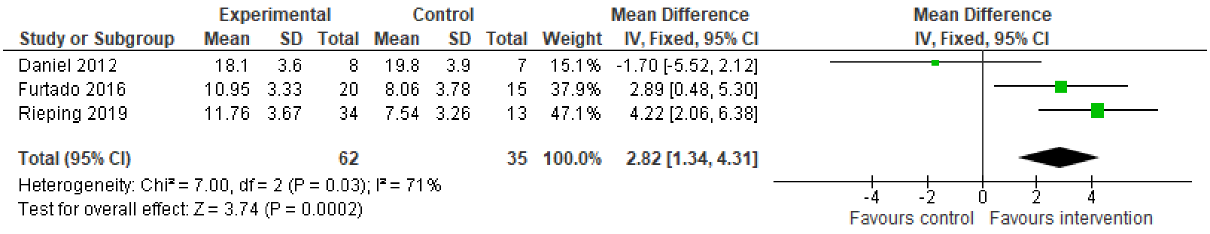

Figure S6: 30 second chair stand

	Experimental			Control				Mean Difference
**Study or Subgroup**	**Mean**	**SD**	**Total**	**Mean**	**SD**	**Total**	**Weight**	**IV, Fixed, 95% CI**
Daniel 2012	11.63	4	8	15	5.7	7	10.2%	-3.37 [-8.42, 1.68]
Furtado 2016	9.3	4.24	20	6.2	3.91	15	35.3%	3.10 [0.39, 5.81]
Rieping 2019	9.6	3.32	34	6.85	3.46	13	54.4%	2.75 [0.56, 4.94]
**Total (95% CI)**			**62**			**35**	**100.0%**	**2.25 [0.64, 3.86]**

Heterogeneity: Chi² = 5.33, df = 2 (P = 0.07); I² = 62%
Test for overall effect: Z = 2.73 (P = 0.006)

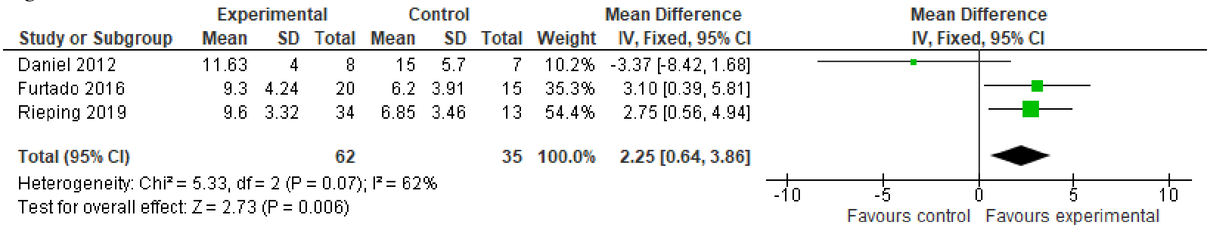

Figure S7: Activities of Daily Living

	Intervention			Control				Std. Mean Difference
**Study or Subgroup**	**Mean**	**SD**	**Total**	**Mean**	**SD**	**Total**	**Weight**	**IV, Random, 95% CI**
Furtado 2016	21.55	3.59	20	22.33	4.28	15	26.3%	-0.20 [-0.87, 0.48]
Furtado 2020	1.25	1.78	21	1.87	1.64	19	27.1%	-0.35 [-0.98, 0.27]
Rieping 2019	26.73	4.17	15	22.62	4.56	13	24.2%	0.92 [0.13, 1.70]
Venturelli 2010	34.8	14.9	12	19.3	11.9	11	22.4%	1.10 [0.21, 1.99]
**Total (95% CI)**			**68**			**58**	**100.0%**	**0.32 [-0.40, 1.04]**

Heterogeneity: Tau² = 0.39; Chi² = 11.48, df = 3 (P = 0.009); I² = 74%
Test for overall effect: Z = 0.88 (P = 0.38)

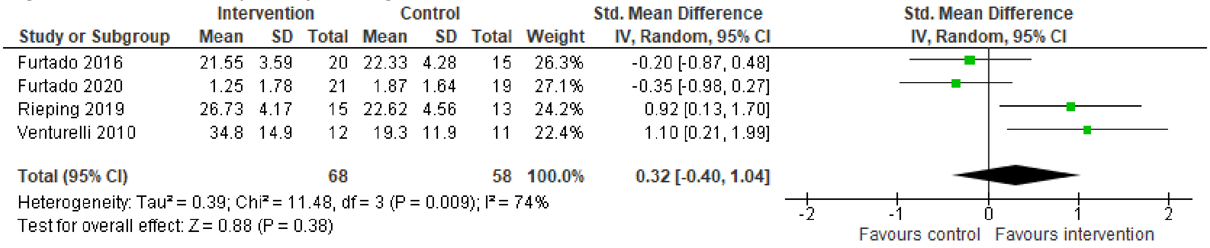

Figure S8: Falls Efficacy

	Intervention			Control				Std. Mean Difference
**Study or Subgroup**	**Mean**	**SD**	**Total**	**Mean**	**SD**	**Total**	**Weight**	**IV, Random, 95% CI**
Furtado 2016	17.9	8.08	20	22.46	10.44	15	18.6%	-0.49 [-1.17, 0.19]
Furtado 2020	32.08	15.43	21	39.67	20.87	19	20.3%	-0.41 [-1.04, 0.22]
Ikai 2017	134.1	11.6	28	120	33.3	28	23.6%	0.56 [0.02, 1.09]
Kim 2015	21.2	5.9	15	21.9	6.5	15	17.6%	-0.11 [-0.83, 0.61]
Rieping 2019	28.12	17.63	34	27.92	15.3	13	19.9%	0.01 [-0.63, 0.65]
**Total (95% CI)**			**118**			**90**	**100.0%**	**-0.06 [-0.46, 0.34]**

Heterogeneity: Tau² = 0.10; Chi² = 7.83, df = 4 (P = 0.10); I² = 49%
Test for overall effect: Z = 0.29 (P = 0.77)

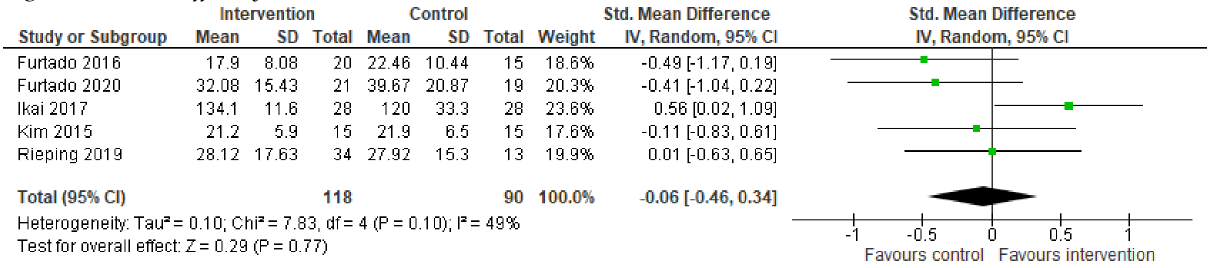

Supplement: Supplementary file 1 [file ijerph-18-01902-s001.pdf]
